# 

**Published:** 1905-05

**Authors:** 


Sixtieth Year.

VOL. LX.
WHOLE NUMBER,
DCCII

MAY, 1905.

New Series.

VOL. XLIV.

No. 10.

### BUFFALO MEDICAL JOURNAL

Established 1845 by AUSTIN FLINT, M. D.

LIBRARY
SURGEON GENERAL'S OFFICE
MAY -1 1905

EDITOR:
WILLIAM WARREN POTTER, M. D.

ASSISTANT EDITORS:

WILLIAM C. KRAUSS, M. D. NELSON W. WILSON, M. D.

ASSOCIATE EDITORS:

JAMES WRIGHT PUTNAM, M. D. ERNEST WENDE, M. D.

JOHN PARMENTER, M. D. JOHN A. MILLER, PH. D.

HARVEY R. GAYLORD, M. D. MAUD J. FRYE, M. D.

For terms and other information relating to the Journal, see page vi.

